# Technology innovation for infectious diseases in the developing world

**DOI:** 10.1186/2049-9957-1-2

**Published:** 2012-10-25

**Authors:** Anthony D So, Quentin Ruiz-Esparza

**Affiliations:** 1 Program on Global Health and Technology Access, Sanford School of Public Policy and Duke Global Health Institute, Duke University, 302 Towerview Drive, CB# 90312, Durham, North Carolina, 27708, USA

**Keywords:** Innovation, Technology, Access, Policy, Public health, R&D collaboration, R&D, Business model, Open source, Open innovation

## Abstract

Enabling innovation and access to health technologies remains a key strategy in combating infectious diseases in low- and middle-income countries (LMICs). However, a gulf between paying markets and the endemicity of such diseases has contributed to the dearth of R&D in meeting these public health needs. While the pharmaceutical industry views emerging economies as potential new markets, most of the world’s poorest bottom billion now reside in middle-income countries--a fact that has complicated tiered access arrangements. However, product development partnerships--particularly those involving academic institutions and small firms--find commercial opportunities in pursuing even neglected diseases; and a growing pharmaceutical sector in BRICS countries offers hope for an indigenous base of innovation. Such innovation will be shaped by 1) access to building blocks of knowledge; 2) strategic use of intellectual property and innovative financing to meet public health goals; 3) collaborative norms of open innovation; and 4) alternative business models, some with a double bottom line. Facing such resource constraints, LMICs are poised to develop a new, more resource-effective model of innovation that holds exciting promise in meeting the needs of global health.

## Multilingual abstracts

Please see Additional file 1 for translations of the abstract into the six official working languages of the United Nations.

## Background

Infectious diseases remain a significant contributor to the burden of disease in low- and middle-income countries (LMICs). Leading communicable diseases from AIDS, tuberculosis and malaria to diarrheal diseases, measles, and lower respiratory infections claim upwards of eleven million lives in these countries each year [1]. The burden falls disproportionately not only on some countries, but also on vulnerable parts of the population. Notably, 95 percent of deaths from respiratory infections and 98 percent of deaths from diarrheal diseases occur in LMICs [2]; and diarrhea, pneumonia, measles, and malaria take many lives of children under five. Similarly, infectious diseases like schistosomiasis, hookworm and malaria contribute to anemia, worsening outcomes both of mother and child in pregnancy, while syphilis also adversely affects neonatal mortality. As for diseases that make up substantial portions of global disease burden--HIV/AIDS, tuberculosis and malaria—over 95 percent of the deaths caused by each of these diseases are also in LMICs. The toll of infectious diseases comes in mortality and morbidity, lost work productivity and economic losses, and the drag effect on those trapped or tipped into poverty by illness.

The attainment of the Millennium Development Goals (MDGs) is closely entwined with progress in reducing the burden of infectious diseases. MDG 6 focuses on combating HIV/AIDS, malaria and other diseases while steps towards meeting MDG 4 (reducing child mortality), MDG 5 (improving maternal health) and MDG 7C (improving basic sanitation and sustainable access to safe drinking water) also relate to the treatment of infectious diseases [3]. MDG 8E (providing access to affordable essential drugs in developing countries in cooperation with pharmaceutical companies) and MDG 8F (making available benefits of new technologies, especially information and communications, in cooperation with the private sector) not only align with these goals, but also suggest instrumental means for accomplishing them [4].

From triple therapy for AIDS and directly-observed therapy for TB to oral rehydration salts and vaccines for childhood killers like diarrhea and pneumonia, the past decade has witnessed significant advances. Between 2000 and 2010, 45 global health technologies were introduced for use in resource-limited settings, and the current R&D pipeline for global health includes 365 medical products at various stages of development [5]. Nevertheless, the unfinished agenda will require further technology innovation. Health technologies diagnose, prevent and treat disease; reduce the risk of disease such as through improved sanitation; mitigate health outcomes (for example, by combating malnutrition); and ensure better delivery of these interventions.

Still many of these technologies remain out of reach to millions who might benefit. For LMICs as a group, the annual health expenditure per capita is just under US$200 as of 2010 [6]. While middle-income countries together have seen a substantial rise in per capita annual health expenditure--from around US$50 in 1995 to about US$220 in 2010--health spending for households in low-income countries, overall, has remained much lower, climbing just US$16 over the same period to US$26 per capita. Though the price of antiretroviral (ARV) therapy has fallen dramatically by 99 percen**t** over the past decade [7], less than a quarter of those in need of ARVs actually received treatment in 2010 [8]. This leaves at least 29.5 million people living with HlV residing in low- and middle-income countries still without treatment, based on 2009 prevalence data [9,10]. Such technologies can also come at considerable cost to these health systems. Out-of-pocket payments remain the primary source for covering the cost of medicines in low- and middle-income countries [11]. 

Applying a systems thinking perspective, more might be done to reshape the enabling environment for innovating such health technologies. Meeting these twin goals of innovation and access is key to bringing technologies from bench to bedside. Focusing on the pharmaceutical value chain might offer insights on how best to ensure technology innovation and access appropriate to disease-endemic countries. Delivering effective artemisinin combination therapy for malaria provides a case example where several interventions along the value chain shape the availability and affordability of this treatment.

"***Delivering Existing Health Technologies to Those in Need--Artemisinin Combination Therapy for Malaria***"

*The Affordable Medicines Facility-Malaria (AMFm) has sought to stem irrational treatment approaches to malaria. AMFm worked to negotiate for lower prices of artemisinin combination treatment (ACT) with manufacturers, subsidize the purchase of ACTs through copayments, and support interventions that encourage rational use of ACTs. By sharply reducing the retail prices of ACTs, the initiative hoped to displace oral artemisinin monotherapies and other medicines, such as chloroquine and sulfadoxine-pyrimethamine, to which resistance had emerged. UNITAID, the Gates Foundation and DFID supported the piloting of this intervention with US$216 million while the Global Fund complemented this, committing up to US$127 million towards supporting interventions for scaling up ACT use effectively [*[12]*]*.

*The premise and preliminary findings behind this pilot illustrate the complexity of delivering even existing innovations to those in need. Early findings from the Health Action International pricing survey in six African countries showed that the AMFm price bested the originator brand and the lowest-priced generic, approaching but not yet consistently beating the prices of irrational alternatives, like chloroquine and sulfadoxine-pyrimethamine [*[13]*]. In less than a year though, six out of eight pilot countries met or exceeded benchmarks for availability, price and market share of quality-assured ACTs, both in rural and urban areas [*[14]*]. Efforts in Tanzania showed that accredited drug dispensing outlets could complement these upstream interventions by increasing access to and dispensing of subsidized artemisinin combination therapy [*[15]*]. However, the volatility in artemisinin supply upstream has caused significant price fluctuations in the pricing of the active pharmaceutical ingredient of this drug. Though it may not offset entirely the greater demand for artemisinin from an expanded AMFm, the anticipated advent of artemisinin, sourced from microbial production, by late 2012 is a technology that may help stabilize and secure the supply of this critical drug for ACT [*[16]*]*.

Innovation can take several forms. For disease-endemic countries, the technology challenge may not only be one of novel invention, but also local adaptation of an existing technology. Such adaptation might be to target locally endemic strains, as for meningococcal or pneumococcal vaccines, where the introduction of such technologies in LMICs has lagged. Or such health technologies might require being lyophilized, kept in cold chain storage, or perhaps soon stabilized in silk films [17] for transport in tropical climates. Or perhaps crucially, technology adaptation to meet the resource constraints in disease-endemic countries--where trained health personnel or health care infrastructure may be wanting--might be required.

Of course, encouraging innovation for disease-endemic countries is not necessarily the same as engaging disease-endemic countries in the process of innovating health technologies for their settings. Looking at industry-supported phase 3 clinical trials conducted by the twenty largest U.S.-based pharmaceutical companies, a third of such studies are now being conducted solely outside of the United States, and a majority of study sites now reside outside of the United States [18]. Much of this globalization of clinical research is to low- and middle-income countries. Shifting that involvement upstream--from conducting clinical trials in disease-endemic settings to working on the bench science--would also mark progress in building the innovation capacity of disease-endemic countries.

In considering access, several related dimensions--each corresponding to a different part of the value chain of delivering technologies--matter. The primary focus for technology innovation is therapeutic access, which refers to whether diagnostics, drugs or vaccines are under research and development or not in the pipeline. Financial and structural access issues also play important roles in enabling diffusion of such technologies. The failure to deliver existing technologies, like AIDS treatment or oral rehydration salts for children with diarrhea, illustrates the challenges of financial and structural access barriers, respectively. Therapeutic access refers to how well the R&D pipeline works, financial access to the market, and structural access to the delivery system.

## Discussion

### Bridging the gulf between markets and disease endemicity

By deaths and DALYs, the focus on HIV/AIDS, tuberculosis and malaria on the global health landscape is understandable: the “Big Three” diseases account for over 4.3 million deaths per year [19]. While the burden of disease falls disproportionately still on low- and middle-income countries, there remains a significant paying market in industrialized countries as well. Boosted by public funding for these diseases, private sector interest is also correspondingly greater.

In a survey of R&D projects focused on neglected diseases, BioVentures for Global Health found 218 R&D projects on AIDS, tuberculosis and malaria--over four times the number of projects on diarrheal diseases (including rotavirus, cholera, typhoid fever, shigellosis, enterotoxigenic E. coli) and pneumococcal disease. By contrast, these other diseases of poverty, specifically various causes of diarrheal disease and pneumococcal infection, claim 3.8 million deaths each year [20]. The number of projects cannot tell the full story: the state-of-the-art and the technical feasibility of next steps vary by disease. Still the difference should provoke reflection on how priorities are set.

Traditional distinctions among Type I diseases (those endemic in both North and South, but with a sizable paying market in the North) and Type II diseases (also endemic globally, but disproportionately so in developing countries, such as AIDS and tuberculosis) and Type III diseases (endemic only in developing countries) depend on the size of potential paying markets for these diseases. Such distinctions may help bound likely contributions and interest from the private sector in these areas of pharmaceutical discovery and R&D. Where there are no paying markets, market failures result.

Bridging this gulf, public sector investments can play an important role in driving this innovation. Between 2007 and 2010, the G-Finder survey found that 97 percent of the research funding backing neglected disease research projects originates from high-income countries [21]. Nearly 64 percent of all funders’ monies comes from the United States. Most publicly funded product development partnerships concentrate their missions around a unifying disease and technology focus, but alternative approaches spanning a cluster of diseases raise the prospect of sharing a common technology platform.

As a market, emerging economies have caught the attention of the global pharmaceutical industry. On the one hand, the industry eyes the growing middle and upper classes of these emerging economies as potential paying customers. On the other hand, 960 million of the bottom billion in the world live in middle-income countries. This is in sharp contrast to two decades ago when over 90 percent of the poorest of the poor lived in low-income countries. Most of these poor people live in countries such as India, Pakistan, Indonesia, and Nigeria, which have graduated from low- to middle-income status [22].

This has implications for pharmaceutical innovation and access. For example, in establishing tiering schemes that give preferential access, from licensed technology to product prices, this tension has made firms reluctant to offer such breaks to middle-income countries. This is reflected in the challenges that the Medicines Patent Pool faces in recruiting companies to license voluntarily their HIV/AIDS drugs for generic production as part of fixed-dose combinations. Similarly, the anchoring of the GlaxoSmithKline-initiated Pool for Open Innovation Against Tropical Neglected Diseases and WIPO Re:Search Consortium--both efforts to pool building blocks of knowledge and to license them royalty-free to those working on neglected diseases--places limits on geographic coverage to only least developed countries as a starting condition.

This underscores the different circumstances facing emerging economies and other developing countries. The stark reality is that less than a quarter of all biomedical research publications and less than a third of all clinical trials in Africa even relate to diseases that comprise nearly 50 percent of the burden of disease on the continent [23]. The same study found that both the research institutions most productive in publishing journal articles and filing patents were concentrated in a few countries in Africa (notably South Africa, Nigeria and Egypt). Examining patterns of collaboration on biomedical publications, over three-quarters of these journal articles were co-authored with collaborators, but only 5.4 percent engaged institutions in more than one African country while the preponderant majority of articles involved collaborators in Europe or the United States. This pattern of collaboration, in part, motivated the formation of the African Network for Drugs and Diagnostics Innovation, with its focus on intra-African coordination and collaboration on R&D.

### Opportunity of technology innovation under resource-constrained settings

Mobilizing public and private sector resources, product development partnerships (PDPs) have stepped in to address the market’s failure to bring forward treatments for neglected diseases. A study of 63 neglected disease projects at the end of 2004 tells an interesting story [24]. Half of these projects were conducted by multinational firms, invariably on a “no profit-no loss” basis. Projects from the other half were conducted on a commercial basis by small-scale entities: small and medium enterprises, developing country firms and academic research institutions. Arguably, these groups viewed the opportunity costs quite differently than did the multinational corporations. This may be an important insight in targeting incentives for companies to help overcome the market’s failure.

A survey of product developers engaged in drug and vaccine R&D for neglected diseases suggested, however, that only 40 percent of such projects involved a PDP [25], with the majority going forward without a PDP partner. These included strong involvement of academic institutions, particularly in the study of neglected tropical diseases. Less than 3 percent of biotechnology companies globally participate in neglected disease R&D, but this still comes to more than 100 firms. Thirteen of the twenty largest pharmaceutical companies are involved in such projects. Multinational drug firms have also begun to shift their patterns of R&D, with recent boosts in approvals of new drugs targeting orphan diseases [26]. In some respects, orphan diseases and neglected diseases may be two sides of the same coin. By value, both face small markets: orphan diseases with small numbers of patients, but treatments that may command high prices in industrialized countries; neglected diseases with millions of patients, but hopes for very low cost treatments per episode. Facing the looming cliff of patent expirations, some of these firms might also be seeing the opportunity costs of smaller markets differently than in the past.

Emerging economies may play an increasingly strategic role in this space. Already India and China are making significant investments in domestic R&D efforts. Among BRICS countries (Brazil, Russia, India, China, and South Africa), foreign assistance to other developing countries has risen at near double-digit rates from 2005 to 2010 [27].

For Brazil, health has been a significant component of the country’s foreign assistance budget. Brazil contributed approximately US$130 million to WHO and PAHO between 2005 and 2009 [28] and pledged US$20 million to the Global Alliance for Vaccines and Immunizations over a twenty-year period [29]. Brazil also provided over US$37 million to UNITAID between 2006 and 2011; and in May 2011, Brazil enacted a law that donates US$2 to UNITAID per international flight, a contribution estimated to grow into a US$12 million commitment annually [30]. Brazil has also initiated a public-private partnership to transfer ARV production technology to Mozambique [31]. Together with the government of Mozambique and the Vale Foundation (the philanthropic arm of a Brazilian mining firm with operations in Mozambique), Brazil’s Institute of Technology in Pharmacos (Farmanguinhos) provided US$23 million to help build an ARV manufacturing plant [32]. Once operational, the plant will produce five ARV drugs and other pharmaceuticals, including a pain reliever and a drug for high blood pressure. In another example of South-South technology transfer, Farmanguinhos, with the Indian drug manufacturer Cipla, also partnered with the Drugs for Neglected Diseases Initiative (DND*i*), a product development partnership, to bring to market a new fixed-dosed artemisinin-based combination treatment, ASMQ, the first ACT with a three-year shelf life in tropical climates [33]. Farmanguinhos and Cipla agreed in 2008 to manufacture and provide ASMQ to the public sector in developing countries at cost (with a target price of US$2.50 per full adult treatment).

### Technology innovation in disease-endemic countries

Innovation for neglected diseases, more often than not, is viewed less as an exemplar to emulate and more as an exception. Just because pharmaceutical and biotechnology firms contribute to neglected disease projects, some might argue that this does not translate to new models of R&D collaboration from which broader, more generalizable lessons might be derived for more commercially viable therapeutic areas.

The perception is, by value, that markets in LMICs are considered small; and the diseases, are typically classified as Type II or III. After all, over three-quarters of global expenditures on pharmaceuticals are spent on 16 percent of the world’s population living in high-income countries [34]. However, the patent cliff faced by multinational pharmaceutical firms, the burden of non-communicable diseases requiring treatment in low- and middle-income countries, the availability of public sector funding and philanthropic capital, and the growing footprint of indigenous innovation in the pharmaceutical sector of emerging economies might prompt rethinking this view.

Spurred initially by the struggle for affordable medicines to treat HIV/AIDS, a decade-long policy process--beginning with the WHO’s Commission on Intellectual Property, Innovation and Public Health [35], continuing with the World Health Assembly’s adoption of the Global Strategy and Plan of Action on Public Health, Innovation and Intellectual Property [36], and leading up to the recently released recommendations of the WHO’s Consultative Expert Working Group on Research and Development: Financing and Coordination [37] —has sought to reshape the way in which health technologies come to market in resource-limited settings.

Several developments may shape and nurture the direction this approach to innovation emerging from developing countries takes. Important elements of this enabling environment include 1) access to building blocks of knowledge; 2) strategic use of intellectual property and innovative financing to meet public health goals; 3) collaborative norms of open innovation; and 4) alternative business models, some with a double bottom line.

Access to the building blocks of knowledge is key to innovation and technology transfer. Journal subscription costs pose barriers to accessing the latest developments in research. In response, WHO has supported the Health InterNetwork Access to Research Initiative (HINARI). Working with publishers, HINARI provides tiered access to journal articles for low- and middle-income countries. This approach has been imperfect, with all BRICS countries, Indonesia, Thailand and other middle-income countries ineligible for the discounted subscription arrangements despite sizable poor populations and under-resourced research institutions in these countries.

While such voluntary agreements provide work-around solutions to improved access to the research literature, industrialized countries have made significant strides to advancing an open access model for sharing journal publications. Departing from the traditional model of subscription-supported publication, open access journals raise revenues from other sources, ranging from endowments and membership dues to advertising and upfront submission or publication fees. As a result, published articles in open access journals are made available freely on-line without subscription barriers. A growing number of leading universities, from Harvard University to the Massachusetts Institute of Technology, have also established institutional, open access repositories where faculty may deposit their publications. Funders like the NIH and the Wellcome Trust have taken steps to require grantees to make available their journal publications in publicly accessible archives. The model of open access publication not only may have more universal applicability, both North and South, but also enables users to assemble relevant publications from multiple journals, without the barrier of subscription costs to each journal.

The past decade has witnessed shortfalls in the supply of influenza vaccine to meet the H1N1 pandemic and heightened concerns over the spread of emerging infectious diseases like SARS and avian flu. Not wishing to be last in queue for a vaccine or treatment, developing countries have sought assurances from WHO’s Global Influenza Surveillance and Response System (GISRS) that the sharing of virus samples would result not just in vaccines for industrialized countries, but also affordable access for such technologies in their settings as well. The Pandemic Influenza Preparedness Framework lays out a standard material transfer agreement, a system for benefit sharing and for contributions from pharmaceutical manufacturers and public health researchers, and hortatory measures encouraging Member States to urge manufacturers to set aside vaccines for influenza strains with pandemic potential for stockpiling and use by developing countries, to engage in technology transfer efforts, and to make such vaccines and antivirals available under tiered pricing arrangements [38]. Whether these measures will suffice in the event of a pandemic will surely be tested in the years ahead.

Anticipating the need to scale up this technology, WHO has provided seed grants to 11 manufacturers in low- and middle-income countries to establish or enhance their capacity to produce pandemic influenza vaccine. The Netherlands Vaccine Institute was not only enlisted to provide training, but also to support an influenza vaccine technology platform or hub to facilitate the transfer of technology to these countries. Building upon a “robust and transferable monovalent pilot process for egg-based inactivated whole virus influenza A vaccine production,” next steps are being planned for work under this technology platform [39]. Such technology platforms might be potentially developed for other diagnostic and therapeutic areas to accelerate innovation.

The strategic management of intellectual property rights is central to securing access to these building blocks of knowledge [40]. Even when publicly funded, patented inventions may not be easily accessible by other researchers or for use in disease-endemic countries. There may be even less incentive to share when such inventions are proprietary and privately funded. However, especially in the neglected disease space, both tiering and pooling arrangements have afforded greater access to needed research inputs. By tiering, preferential discounts or even royalty-free access to research inputs is provided, often bounded by field of use or geography. For AIDS drugs and many vaccines, tiered pricing arrangements offer price breaks to resource-limited countries. Further upstream, many neglected disease projects benefit from tiered licensing arrangements, whereby access to compounds otherwise inaccessible due to their proprietary nature is provided. The R&D pathway for repurposing existing drugs can be shortened considerably when such access is coupled with pre-clinical and even clinical data on these compounds. Under pooling arrangements, the transaction costs of bringing together needed inputs for research are lowered and cross-licensing enabled.

With varying degrees of resulting access and other forms of success, a patchwork of ad hoc tiering and pooling arrangements have emerged. Both policy instruments have their place in ensuring broader access to research inputs. Collaborating with the Medicines for Malaria Venture, GlaxoSmithKline’s release of the chemical structures and assay data of over 10,000 compounds with activity against the malaria parasite, *Plasmodium falciparum,* is such an example. This deposit into the pool of the European Bioinformatics Institute’s ChEMBL database and the U.S. NIH PubChem database provides wide access under a Creative Commons CC0 license (work dedicated to the public domain with waiver of copyright) [41]. Tiering and pooling often work in tandem. The Medicines Patent Pool, the Pool for Open Innovation Against Neglected Tropical Diseases, and the WIPO Re:Search Consortium all represent pooling arrangements, each with different tiered access conditions on inputs into the pool. Some work by aggregating research inputs upstream in the R&D pipeline, and others recruit patented drugs downstream in the R&D pipeline. For example, the Medicines Patent Pool’s mission is to secure voluntary licenses from pharmaceutical companies of HIV/AIDS drugs that might be used in new fixed-dose combinations or pediatric formulations. In so doing, the generic licensing of such combinations is meant to stir greater competition and thereby innovation and affordability of such treatments. Negotiating licenses, limited by field and geography, has proven challenging. To date, the only license with a company for AIDS medicines has been with Gilead. This license restricts manufacturing to India, but extends access to resulting products to a wider range of countries (though still excluding several middle-income countries) than under previous arrangements [42].

As with open access initiatives, funders can set norms supportive of sharing knowledge and also lower the risks of crossing the valley of death from pre-clinical to clinical testing. The NIH recently launched the National Center for Advancing Translational Sciences (NCATS). Created to “catalyze the generation of innovative methods and technologies” that might bring diagnostics and therapeutics to first-in-human trials, NCATS provides a spectrum of intramural and contracted services that enable small firms and academic research institutions to secure needed preclinical support [43]. Funders can also invest their philanthropic capital in ways to ensure more affordable access in exchange for non-diluting cash for biotechnology start-up companies. With Gates Foundation support, the University of California, Berkeley extended co-exclusive licenses for the microbial synthesis of artemisinin--a key antimalarial drug--to Amyris Biotechnologies and the Institute for One World Health [44]. The University made the license royalty-free for malaria indications in exchange for Amyris Biotechnologies’ commitment to produce artemisinin at no profit for treating malaria in the developing world. Amyris, in return, also received substantial philanthropic capital support (US$12 million) from the Gates Foundation. This enabled Amyris to develop proof of concept on its microbial synthesis process, which also has a dual market application for synthesizing biofuels.

Establishing collaborative norms of open innovation has deep roots in public sector funding of science. The Wellcome Trust and the U.S. National Institutes of Health (NIH) engaged leading centers involved in the Human Genome Project to agree to the Bermuda Rules, whereby investigators pledged to deposit gene sequences of every 1000 base pairs within 24 hours of completion into GenBank [45]. The intent was not only to encourage sharing of data, but also to prevent unnecessary patenting through defensive publishing. NIH has also issued guidance to grantees for the “timely release and sharing of final research data” for others to use [46] and for minimizing unnecessary encumbrances on the dissemination of publicly funded research tools [47].

Increasingly, the pharmaceutical sector has recognized the value of open innovation [48]. From Merck’s early efforts to place expressed sequence tags into the public domain to corporate participation in the Single Nucleotide Polymorphisms Consortium, pharmaceutical firms have understood the need to harness ideas from outside their walls to fuel in-house R&D innovation. The potential application for open innovation is most evident for emerging infectious diseases, where the patenting process might be outpaced by the speed of a spreading pandemic. The need for pooling patents on SARS to enable non-exclusive licensing anticipated the potential problems of intellectual property holdings on developing a diagnostic or treatment for the illness. However, the SARS epidemic came and went before the patent pool could be launched, highlighting the potential value of open innovation norms for emerging diseases.

Taking this a step further, fledgling efforts to conduct open source innovation in biomedicine have also begun. By contrast, open source innovation involves openness and transparency with the goal of shared research collaboration, but also prevents third parties from acquiring proprietary rights over what is generated by the community. Notably, India’s Council for Scientific and Industrial Research has embarked on the Open Source Drug Discovery (OSDD) initiative, initially focused on TB drugs. Using a web-based platform, hundreds of volunteers at a network of universities, both in India and elsewhere, have collaborated on re-annotating the *Mycobacterium tuberculosis* genome. Their collective efforts made this possible in just four months. Supported with government funding, participants submit projects for open peer review, contribute to on-line efforts under a system of microattribution, and agree to sharing their work under a click-wrap license. In not pursuing a conventional path to drug discovery, OSDD hopes to “bring down the cost of drug discovery significantly by knowledge sharing and constructive collaboration” and “to discover new chemical entities and to make them generic as soon as they are discovered, thus expediting the process of drug discovery” [49].

### Summary

For many neglected diseases, the case for an alternative business model is clear. On the demand side, the need to provide affordable health technologies responsive to public health needs and the local context of low- and middle-income countries is urgent. On the supply side, high volume, closer to marginal cost pricing might match this need. Fortunately, there is growing capacity among developing countries to respond. Developing country vaccine manufacturers already supply 64 percent of vaccines procured by UNICEF [50]; and more than 80 percent of annual purchase volumes of antiretroviral drugs destined for low- and middle-income countries come from Indian generic manufacturers [51].

Bridging the supply and demand side, there is need for a more efficient model for R&D innovation. This will likely require innovation of both products and processes. Described by various names, the idea of *jugaad* innovation, a Hindi word meaning “an innovative fix; an improvised solution born from ingenuity and cleverness; resourceful” captures, in part, the spirit of such efforts [52]. Others have applied the descriptor, “frugal innovation” [53]. However, it is important not to connote that such innovation will come as a quick fix. Nor will it come out of a sense of thriftiness alone, without a deeper understanding of the effective use of resources. The enabling conditions for such innovation must be carefully cultivated and nurtured, and this may require piloting new models for collaborative R&D efforts--sharing resources, risks and rewards more effectively [54].

Working under the constraints of a resource-limited environment, such innovation must be resource-effective, but not substandard. This efficient use of resources is reflected in the Innovation Efficiency Index scoring of countries like China and India compared to developed countries. This index compares outputs from innovation against the constraint of available inputs for innovation in a country, and by this measure, China and India come out first and second in the world [55]. Perhaps fittingly, some have suggested that such innovation embraces Mahatma Gandhi’s tenet of getting “more from less for more people,” [56] best captured in his quote: “Earth provides enough to satisfy every man’s need, but not every man’s greed” [57].

The enabling environment for resource-effective innovation will likely accelerate not only health technologies for infectious diseases, but also for the growing burden of non-communicable diseases. Technologies like echocardiography have clinical value whether the valvular heart disease traces to rheumatic fever, drugs or atherosclerosis. The process of technology transfer, the clinical trial platforms, and the training of scientists also build towards the common purpose of bringing these new health technologies from bench to bedside.

What is exciting for global health is that the world--both North and South--has much to benefit from these new approaches to innovation. Under increasing budget constraints themselves, industrialized countries might welcome cost-effective interventions born out of the genius of innovation under resource constraint. Such innovation might be potentially disruptive, in that a product targeted at the base of the economic pyramid--an initial market marginalized by competitors--might migrate “up market” displacing established technologies [58]. From point-of-care diagnostics to more affordable drugs and vaccines, the necessity to bring more appropriate and affordable health technologies may indeed spark a new approach to innovation.

## Abbreviations

ACT: Artemisinin-combination therapies; AIDS: Acquired immune deficiency syndrome; AMFm: Affordable Medicines Facility - malaria; ARV: Antiretrovirals; ASMQ: An ACT fixed-dose combination of artesunate and mefloquine; CC0: The “no rights reserved” Creative Commons licensing; ChEMBL: A database of drug-like small molecules affiliated with the European Molecular Biology Laboratory; BRICS: Brazil, Russia, India, China, and South Africa; DALY: Disability-adjusted life years; DFID: United Kingdom Department for International Development; DND*i*: Drugs for Neglected Diseases Initiative; G-FINDER: Global Funding of Innovation for Neglected Diseases; GISRS: Global Influenza Surveillance and Response System; H1N1: A subtype of the influenza A virus, also called swine flu; HINARI: Health InterNetwork Access to Research Initiative; HIV: Human immunodeficiency virus; LMIC: Low- and middle- income countries MDG:Millennium Development Goals NCATS:U.S. National Center for Advancing Translational Science NIH:U.S. National Institutes of Health OSDD:Open Source Drug Discovery PAHO:Pan-American Health Organization PDP:Product development partnership R&D:Research and development SARS:Severe acute respiratory syndrome TB:Tuberculosis UNAIDS:Joint United Nations Programme on HIV/AIDS UNITAID:An intergovernmental organization that works through market interventions to make life-saving products better and more affordable UNICEF:United Nations Children’s Fund U.S.:United States US$:U.S. dollars WHO:World Health Organization WIPO:World Intellectual Property Organization.

## Competing interests

The Program on Global Health and Technology Access, of which ADS serves as the Program Director and QRE an Associate in Research, provides consultancy services on intellectual property management for a product development partnership, the Drugs for Neglected Diseases Initiative (DND*i*).

## Authors’ contributions

ADS led in the conceptualization and design behind the overall organization of the manuscript and substantively contributed to the collection of information and data presented in this manuscript. ADS also substantially wrote the majority of the manuscript and also gave the approval of the final submitted version of the manuscript. QRE contributed to the analysis and research that provided the evidence and examples given in this paper. QRE also assisted ADS with the drafting of the manuscript. All authors read and approved the final manuscript.

## Authors’ information

ADS is a Professor of the Practice of Public Policy and Global Health at Duke University in the Sanford School of Public Policy. He is also the founder and the Director of the Program on Global Health and Technology Access. Key focus areas of the Program's interdisciplinary work include developing alternative biopharmaceutical R&D models from precompetitive collaboration to public financing of R&D and evaluating models of innovation and access to health technologies. Dr. So has led the Strategic Policy Unit for ReAct, an independent global network for concerted action on antibiotic resistance; advised the Thematic Reference Group for Innovation and Technology Platforms for Infectious Diseases of Poverty, a working group of WHO’s Special Programme for Research and Training In Tropical Diseases; and served as a member of the U.S. Institute of Medicine's Committee on Accelerating Rare Disease Research and Orphan Product Development. He has authored works on innovation for global health R&D, including a commissioned paper for the Institute of Medicine on “Sharing Knowledge for Global Health” and a paper on “Approaches to Intellectual Property and Innovation that Meet the Public Health Challenge of AIDS” for the Global Commission on HIV and the Law. This year, Dr. So was named one of the Robert Wood Johnson Foundation’s Investigators in Health Policy Research. Previously, Dr. So was Associate Director of the Rockefeller Foundation’s Health Equity program, where he co-founded a cross-thematic program on charting a fairer course for intellectual property rights, shaped the Foundation’s work on access to medicines policy in developing countries, and launched a multi-country program in Southeast Asia, “Trading Tobacco for Health,” focused on enabling countries to respond to the public health challenge of tobacco use. QRE is an Associate in Research for the Program on Global Health and Technology Access, helping lead analyses for the global health policy research projects of the Program.

## Supplementary Material

Additional file 1 Multilingual abstracts in the six official working languages of the United Nations. Click here for file

## References

[B1] Lopez AD Mathers CD Ezzati M Measuring the Global Burden ofDisease and Risk Factors, 1990–2001 Global Burden of Disease and Risk Factors. Chapters 1 and 2 2006 Washington, DC: The International Bank for Reconstruction and Development / The World Bank http://www.dcp2.org/pubs/GBD/21250376

[B2] World Health Organization Mortality and Burden of Disease Estimates for WHO Member States in 2004 2009

[B3] United Nations Millennium Development Goals Report 2011 2011 United Nations: New York http://www.un.org/millenniumgoals/11_MDG%20Report_EN.pdf

[B4] MDG Targets and Indicatorshttp://www.un-ngls.org/spip.php?page=amdg10&id_article=2330

[B5] Global Health Technologies Coalition / Policy Cures Saving Lives and Creating Impact: Why Investing in Global Health Research Works 2012 http://policycures.org/downloads/Saving%20lives%20and%20creating%20impact.pdf

[B6] World Bank Health Expenditure per Capita, data from 1995 to 2010 2010 http://data.worldbank.org/indicator/SH.XPD.PCAP

[B7] Médecins Sans Frontières Untangling the Web of Antiretroviral Price Reductions 2011 14 Geneva, Switzerland: Médecins Sans Frontières

[B8] World Health Organization, UNICEF, UNAIDS Global HIV/AIDS Response 2011 Geneva, Switzerland: Epidemic Update and Health Sector Progress towards Universal Access World Health Organization Department of HIV/AIDS http://www.unaids.org/en/media/unaids/contentassets/documents/unaidspublication/2011/20111130_UA_Report_en.pdf

[B9] UNAIDS AIDSInfo Database: Number of People Living with HIV, 1990 to 2009 2009 http://www.unaids.org/en/dataanalysis/datatools/aidsinfo/

[B10] World Bank Health Expenditure per Captia, data from 1995 to 2010 2010 http://data.worldbank.org/indicator/SH.XPD.PCAP

[B11] Lu Y Hernandez P Abegunde D Edejer T The World Medicines Situation 2011: Medicine Expenditures 2011 3 Geneva, Switzerland: World Health Organization 7

[B12] Adeyi O Affordable Medicines Facility-malaria (AMFm): Achievements to date, UNITAID Consultative Forum 2011 Geneva, Switzerland: UNITAID

[B13] Health Action International Retail prices of ACTs co-paid by the AMFm and other antimalarial medicines: report of price-tracking survey 2011 Geneva, Switzerland: The Global Fund to Fight AIDS, Tuberculosis and Malaria

[B14] Expert Advisory Group on the Affordable Medicines Facility-malaria (AMFm) Review of the AMFm Phase 1 Independent Evaluation Preliminary Report 2012 Geneva, Switzerland: The Global Fund to Fight AIDS, Tuberculosis and Malaria

[B15] Rutta E Kibassa B McKinnon B Liana J Mbwasi R Mlaki W Embrey M Garba M Shekalaghe E Kimatta S Hiiti S Increasing Access to Subsidized Artemisinin-based Combination Therapy through Accredited Drug Dispensing Outlets in Tanzania Health Res Pol Syst 2011 9 22 10.1186/1478-4505-9-22 PMC3141785 21658259

[B16] The William Davidson Institute: Affordable Medicines Facility-malaria (AMFm) Assessment of the effects of the AMFm on market dynamics of API and ACTs at the global level 2012 Ann Arbor, Michigan: William Davidson Institute, The University of Michigan http://www.theglobalfund.org/en/amfm/discussionpapers/

[B17] Zhang J Pritchard E Hu X Valentin T Panilaitis B Omenetto FG Kaplan DL Stabilization of vaccines and antibiotics in silk and eliminating the cold chain Proc Natl Acad Sci 2012 109 30 11981 11986 10.1073/pnas.1206210109 22778443 PMC3409735

[B18] Glickman SW McHutchison JG Peterson ED Cairns CB Harrington RA Califf RM Schulman KA Ethical and Scientific Implications of the Globalization of Clinical Research N Engl J Med 2009 360 8 816 823 10.1056/NEJMsb0803929 19228627

[B19] World Health Organization Mortality and Burden of Disease Estimates for WHO Member States in 2004 2009

[B20] World Health Organization Mortality and Burden of Disease Estimates for WHO Member States in 2004 2009

[B21] Policy C Global Funding for Innovation for Neglected Diseases (G-FINDER) 2011 http://g-finder.policycures.org/gfinder_report/

[B22] Sumner A The New Bottom Billion: What If Most of the World’s Poor Live in Middle-Income Countries? 2011 Washington, DC: Center for Global Development http://www.cgdev.org/files/1424922_file_Sumner_brief_MIC_poor_FINAL.pdf

[B23] Nwaka S Ilunga TB Da Silva JS Rial Verde E Hackley D De Vré R Mboya-Okeyo T Ridley RG Developing ANDI: A Novel Approach to Health Product R&D in Africa PLoS Med 2010 7 6 e1000293 http://www.plosmedicine.org/article/info%3Adoi%2F10.1371%2Fjournal.pmed.1000293#pmed-1000293-g001 10.1371/journal.pmed.1000293 20613865 PMC2893959

[B24] Moran M A Breakthrough in R&D for Neglected Diseases: New Ways to Get the Drugs We Need PLoS Med 2005 2 9 e302 http://www.plosmedicine.org/article/info%3Adoi%2F10.1371%2Fjournal.pmed.0020302 10.1371/journal.pmed.0020302 16138789 PMC1198042

[B25] Ponder E Moree M Developing New Drugs & Vaccines for Neglected Diseases of the Poor: The Product Developer Landscape 2012 Washington, DC: BIO Ventures for Global Health

[B26] Associated Press Innovative drug approvals up sharply in 2011, gov’t increasingly funds infectious disease R&D 2012 Washington, DC: Washington Post

[B27] Global Health Strategies Initiatives Shifting Paradigm: How the BRICS are Reshaping Global Health and Development 2012 New York, NY: Global Health Strategies Initiatives

[B28] Institute for Applied Economic Research Brazilian Cooperation for International Development: 2005–2009 2011 Rio de Janeiro, Brazil: Institute for Applied Economic Research

[B29] GAVI Alliance Brazil: Donor Profiles http://www.gavialliance.org/funding/donor-profiles/brazil/

[B30] UNITAID UNITAID Annual Report 2011: Five Years for Better Health 2011 Geneva, Switzerland: UNITAID

[B31] Technology transfer and establishment of an antiretroviral drugs factory in Mozambique http://www.fiotec.fiocruz.br/institucional/index.php?option=com_content&view=article&id=805:technology-transfer-and-establishment-of-an-antiretroviral-drugs-factory-in-mozambique&catid=126&Itemid=489&lang=en

[B32] AFP Mozambique launches Brazil-funded drugs plant to battle HIV. Yahoo! News 2012 http://news.yahoo.com/mozambique-launches-brazil-funded-drugs-plant-battle-hiv-174318951.html

[B33] Drugs for Neglected Diseases Initiative A Worldwide Public Partnership Makes Available a New, Once-a-Day Fixed-Dose Combination against Malaria 2008 Geneva, Switzlerand: Drugs for Neglected Diseases Initiative http://dndi.org/press-releases/2008/113-a-worldwide-public-partnership-makes-available-a-new-once-a-day-fixed-dose-combination-against-malaria.html

[B34] Lu Y Hernandez P Abegunde D Edejer T The World Medicines Situation 2011: Medicine Expenditures 2011 3 Geneva, Switzerland: World Health Organization

[B35] Commission on Intellectual Property Rights Innovation and Public Health: Public Health, Innovation and Intellectual Property Rights 2006 Geneva, Switzerland: World Health Organization http://www.who.int/intellectualproperty/documents/thereport/ENPublicHealthReport.pdf

[B36] Sixty-First World Health Assembly Global strategy and plan of action on public health, innovation and intellectual property,” WHA61.21, Agenda item 11.6 2008 http://apps.who.int/gb/ebwha/pdf_files/A61/A61_R21-en.pdf

[B37] Consultative Expert Working Group on Research and Development Financing and Coordination: Research and Development to Meet Health Needs in Developing Countries: Strengthening Global Financing and Coordination 2012 Geneva, Switzerland: World Health Organization http://www.who.int/phi/CEWG_Report_5_April_2012.pdf

[B38] WHO Pandemic influenza preparedness Framework for the sharing of influenza viruses and access to vaccines and other benefits 2011 Geneva, Switzerland: World Health Organization Available at: http://whqlibdoc.who.int/publications/2011/9789241503082_eng.pdf

[B39] Hendriks J Holleman M de Boer O de Jong P Luytjes W An international technology platform for influenza vaccines Vaccine 2011 29 Supplement 1 A8 A11 Available at: http://www.sciencedirect.com/science/article/pii/S0264410X11006906 21684431 10.1016/j.vaccine.2011.04.124

[B40] So AD Stewart E Sharing Knowledge for Global Health The U.S. Commitment to Global Health: Recommendations for the Public and Private Sectors 2008 Washington, DC: Appendix F. U.S. Institute of Medicine Committee on the U.S. Commitment to Global Health [ http://www.ncbi.nlm.nih.gov/books/NBK23792/]

[B41] ChEMBL-Neglected Tropical Disease archive ChEMBL-Neglected Tropical Disease archive [ https://www.ebi.ac.uk/chemblntd/]

[B42] Medicines Patent Pool FAQ on the Pool/Gilead Licenses: October 2011--Frequently Asked Questions on the Implications of the Gilead/Medicines Patent Pool License [ http://www.medicinespatentpool.org/licensing/current-licences/the-medicines-patent-poolgilead-licences-questions-and-answers/faq-on-the-poolgilead-licences/]

[B43] Collins F Reengineering translational science: the time is right Sci Transl Med 2011 3 90:90cm17 1 6 [ http://www.nih.gov/about/director/articles/translational_science_07062011.pdf] 10.1126/scitranslmed.3002747 PMC5101940 21734173

[B44] Mimura C Cheng J Penhoet B Perspective: Socially Responsible Licensing, Euclidean Innovation, and the Valley of Death Stanford Journal of Law, Science and Policy 2011 UC Berkeley Public Law Research Paper No. 1928837. [ http://papers.ssrn.com/sol3/papers.cfm?abstract_id=1928837]

[B45] Marshall E Bermuda Rules: community spirit, with teeth Science 2001 291 1192 10.1126/science.291.5507.1192 11233433

[B46] U.S. National Institutes of Health Final NIH Statement on Sharing Research Data, 2003 2003 Bethesda, MD: Notice NOT-OD-03-032. U.S. National Institutes of Health [ http://grants.nih.gov/grants/guide/notice-files/not-od-03-032.html]

[B47] U.S. National Institutes of Health Principles and Guidelines for Recipients of NIH Research Grants and Contracts on Obtaining and Disseminating Biomedical Research Resources: Final Notice Federal Register Notice 1999 [ http://www.ott.nih.gov/policy/rt_guide_final.html]

[B48] Chesbrough HW The Era of Open Innovation Top 10 Lessons on the New Business of Innovation. MIT Sloan Management Review 2011 35 41 (originally published Spring 2003). [ http://scholar.google.com/scholar_url?hl=en&q=http://sloanreview.mit.edu/files/2011/06/INS0111-Top-Ten-Innovation.pdf%23page%3D37&sa=X&scisig=AAGBfm1PbObbGnXtYKHIQDvZFi1_CRc2Dg&oi=scholarr]

[B49] Council for Scientific and Industrial Research Frequently Asked Questions, Open Source Drug Discovery Initiative [ http://www.osdd.net/faq-s]

[B50] Jadhav SS Gautam M Gairola S Emerging markets & emerging needs: Developing countries vaccine manufacturers’ perspective & its current status Biologicals 2009 37 165 168 10.1016/j.biologicals.2009.02.009 19328010

[B51] Waning B Diedrichsen E Moon S A lifeline to treatment: the role of Indian generic manufacturers in supplying antiretroviral medicines to developing countries J Int AIDS Soc 2010 13 35 [ http://www.ncbi.nlm.nih.gov/pmc/articles/PMC2944814/] 10.1186/1758-2652-13-35 20840741 PMC2944814

[B52] Radjou N Prabhu J Ahuja S Jugaad Innovation: Think Frugal, Be Flexible, Generate Breakthrough Growth 2012 San Francisco, CA: Jossey-Bass

[B53] Bound K Thornton I Our Frugal Future: Lessons from India’s Innovation System 2012 London, United Kingdom: Nesta [ http://www.nesta.org.uk/library/documents/OurFrugFuture.pdf]

[B54] So AD Ruiz-Esparza Q Gupta N Cars O 3Rs for innovating novel antibiotics: sharing resources, risks, and rewards Br Med J 2012 344 e1782 10.1136/bmj.e1782 22491076

[B55] INSEAD, World Intellectual Property Organization The Global Innovation Index 2012: Stronger Innovation Linkages for Global Growth 2012 Fontainebleau, France: INSEAD and World Intellectual Property Organization Edited by Dutta S

[B56] Malhotra A Kleiner A Geller LW A Gandhian Approach to R&D Strategy + Business 2010 [ http://www.strategy-business.com/article/10310?pg=all]

[B57] Prahalad C Mashelkar R Innovation’s Holy Grail Harvard Business Review 2010 88 7/8 132 141 [serial online]

[B58] Clayton Christensen Key concepts--disruptive innovation [ http://www.claytonchristensen.com/key-concepts/]

