# Supplementary material for: Technology innovation for infectious diseases in the developing world
Source: Infect Dis Poverty. 2012 Oct 25;1:2. doi: 10.1186/2049-9957-1-2 (PMC3710188; doi:10.1186/2049-9957-1-2)
Supplement: Additional file 1 — Multilingual abstracts in the six official working languages of the United Nations. [file 2049-9957-1-2-S1.pdf]

Translation of the abstract into the six official working languages of the United Nations

إسبارسا-الابتكار التقني للأمراض المعدية في العالم النامي انتوني دي سو وكوينتين رويز

ملخص

تمكين الإبتكار و الوصول إلى التقنيات الصحية لا تزال استراتيجية رئيسية في مكافحة الأمراض المعدية في البلدان المنخفضة
و استيطان مثل هذه الأمراض في نهاية البحث المربحة ساهمت الثغرة بين الأسواق ومع ذلك، (LMICs) و المتوسطة الدخل
و بينما تشهد صناعة الأدوية اقتصادات ناشئة مثل الأسواق الجديدة .و التطوير من أجل تلبية هذه الاحتياجات الصحية العامة
هذه الحقيقة أدت إلى تعقيد --بلدان متوسطة الدخلفإن مليار شخص الأكثر فقراً في العالم يعيشون الآن في الـ ,المحتلمة
--و خاصة التي تضم المؤسسات الأكاديمية و الشركات الصغرى--إلا أن شراكات تطوير المنتج .ترتيبات الوصول المتدرج
يثةوجدت فرصاً تجارية في متابعة حتى الأمراض المهملة؛ و منحنا القطاع الدوائي المتنامي في الدول الصناعية الحد
سيرسم ملامح هذا الابتكار .الأمل في خلق أسس محلية للابتكار (البريكس-البرازيل وروسيا و الهند و الصين و جنوب أفريقيا)
الاستخدام الاستراتيجي للملكية الفكرية و التمويل المبتكر لتحقيق أهداف الصحة (2 الوصول إلى اللبنات الأساسية للمعرفة؛ (1
مواجهة مثل هذه القيود .بعضها ذو خط احتياطي مضاعف ,نماذج أعمال بديلة (4 قواعد تعاونية للابتكار المفتوح؛ و (3العامة؛
فإن الدول منخفضة و متوسطة الدخل تستعد لتطوير نموذج ابتكار جديد و أكثر فعالية للموارد و الذي ,المفروضة على الموارد
حة العالميةيحمل وعوداً مثيرة لتحقيق احتياجات الصـ.

Translated from English version into Arabic by Berlant Tosson, through

TRANSLATORS
WITHOUT BORDERS

发展中国家传染病的技术创新

Anthony D So， Quentin Ruiz-Esparza

摘要

卫生技术的创新能力和可及性仍然是低收入和中等收入国家(LMICs)对抗传染病的主要策略。然而，传染病的支付市场和本土性之间存在矛盾，导致公共卫生需求的研究和发展较为不足。当医药工业将新兴经济体视作潜在的新市场时，世界上最贫困的底层十亿人口的大部分却停留在中等收入国家行列，分层可及的约定变得更加复杂。但产品开发的合作，特别是学术机构和小公司参与的项目，在从事被忽略疾病的防治中获得商机，而金砖四国快速增长的制药行业为技术创新的本土化带来希望。这些创新由下列因素构成：1）知识的构成；2）知识产权和创新资金的战略利用以满足公共卫生目标；3) 开放性创新的合作规范； 4) 可替代的商业模式，其中一些具有双重底线. 面对这些资源限制，低收入和中等收入国家仍准备着新型资源节约型模式的创新，积极履行以全球健康为目标的承诺。

Translated from English version into Chinese by Zhang Zheng-Yan, through

CDC CHINESE CENTER FOR DISEASE CONTROL AND PREVENTION
NATIONAL INSTITUTE FOR PARASITIC DISEASES

Innovations technologiques pour lutter contre les maladies infectieuses dans les pays en développement

Par Anthony D. So et Quentin Ruiz-Esparza

Résumé

L'une des stratégies-clés de la lutte contre les maladies infectieuses dans les pays à faible et moyen revenus (PFMR) consiste à faciliter l'innovation et l'accès aux technologies de la santé. Le gouffre entre les marchés rémunérateurs et le caractère endémique de ces maladies a cependant contribué à l'incapacité des activités de recherche et de développement à répondre à ces besoins en matière de santé publique. Alors que l'industrie pharmaceutique considère les économies émergentes comme de nouveaux marchés potentiels, la grande majorité du milliard d'habitants les plus pauvres de la planète réside désormais dans des pays à moyen revenu, une situation qui complique les dispositifs d'accès progressif. Les partenariats de développement de produits en revanche, en particulier ceux auxquels participent des institutions universitaires et de petites entreprises, pensent que la recherche sur les maladies même les plus négligées représente une opportunité commerciale, tandis que la croissance du secteur pharmaceutique dans les pays du BRICS (Afrique du Sud, Brésil, Russie, Inde et Chine) donne l'espoir d'une base d'innovation indigène. De telles innovations passent par 1) l'accès aux savoirs fondamentaux ; 2) l'utilisation stratégique de la propriété intellectuelle et des financements innovants pour répondre aux objectifs de santé publique ; 3) des normes collaboratives d'innovation ouverte ; et 4) de nouveaux modèles commerciaux, avec parfois un double objectif de résultats. Face à de telles contraintes en termes de ressources, les PFMR sont décidés à développer un nouveau modèle d'innovation, utilisant les ressources plus efficacement, qui devrait répondre aux besoins en matière de santé mondiale.

Translated from English version into French by ecarliez, through

TRANSLATORS
WITHOUT BORDERS

Инновационная технология в лечении инфекционных заболеваний в развивающихся странах

Энтони Ди Со и Квентин Руис-Эспарса

Аннотация

Предоставленная инновация и доступ к медицинским технологиям сохраняет стратегию в инфекционных заболеваниях, полученных от ранений, в странах с доходами ниже среднего уровня. Однако пропасть между прибыльными рынками и эндемичностью существующих заболеваний способствовала отсутствию исследований и разработок для преодоления потребностей общественного здравоохранения. В то время, когда фармацевтическая промышленность рассматривает переходные экономики как потенциально новые рынки, основной миллиард большинства наихудших мировых экономик пребывают в странах со средним уровнем доходов, что является фактом осложнённой возможности систематизации. Тем не менее, компании по разработке продукции, в частности те, которые включают академические учреждения и малые предприятия, используют коммерческие возможности для лечения даже этих игнорируемых заболеваний, и нарастающий фармацевтический сектор в странах БРИК предоставляет надежду для присущей основы инновации. Такая инновация будет сформирована 1) доступом к основам знаний; 2) стратегическим использованием интеллектуальной собственности и передовым финансированием для достижения цели общественного здравоохранения; 3) совместными нормами для открытых инноваций, и 4) альтернативными моделями предприятия, и социально ответственными предприятиями. Столкнувшись с ограничениями ресурсов, страны с доходами ниже среднего уровня намерены разработать новую, более ресурсно-эффективную инновационную модель, которая поддержит существующую перспективу преодоления потребностей мирового здравоохранения.

Translated from English version into Russian by Halyna Maksymiv, through

TRANSLATORS
WITHOUT BORDERS

Innovación tecnológica contra las enfermedades infecciosas en los países en desarrollo

Anthony D. So, Quentin Ruiz-Esparza

Resumen

La innovación y el acceso a las tecnologías sanitarias siguen siendo las estrategias principales en la lucha contra las enfermedades infecciosas en los países de bajos y medianos ingresos (PBMI). Sin embargo, la diferencia entre los mercados de pago y la endemia de dichas enfermedades ha fomentado la escasez de investigación y desarrollo para cubrir esta necesidad de salud pública. Mientras que la industria farmacéutica ve en las economías en desarrollo a nuevos mercados potenciales, la mayor parte de los mil millones de personas más pobres del mundo residen ahora en países de ingresos medios, lo que complica la aplicación de modelos de precios escalonados. Sin embargo, las asociaciones de desarrollo de productos, especialmente entre instituciones académicas y pequeñas empresas, encuentran oportunidades comerciales incluso entre las enfermedades olvidadas, y el crecimiento de la industria farmacéutica en los países BRIC (Brasil, Rusia, India y China) ofrece esperanzas para una base de innovación autóctona. Dicha innovación vendrá determinada por: 1) el acceso a las estructuras básicas del conocimiento; 2) el uso estratégico de la propiedad intelectual y la financiación innovadora para cumplir los objetivos de salud pública; 3) las normas de colaboración en materia de innovación abierta; y 4) modelos de negocio alternativos, algunos de ellos con objetivos dobles. Al enfrentarse a tal limitación de recursos, los países de bajos y medianos ingresos estarán listos para desarrollar un nuevo modelo de innovación más eficaz, que permita abrigar esperanzas en satisfacer las necesidades médicas globales.

Translated from English version into Spanish by Rosa Sanz, through

TRANSLATORS
WITHOUT BORDERS
